# Effects of 6-Hydroxykaempferol: A Potential Natural Product for Amelioration of Tendon Impairment

**DOI:** 10.3389/fphar.2022.919104

**Published:** 2022-07-22

**Authors:** Tsz Ngai Mok, Qiyu He, Xiaoxi Zhang, Tat Hang Sin, Huajun Wang, Huige Hou, Jinghua Pan, Xiaofei Zheng, Zhengang Zha, Jieruo Li

**Affiliations:** ^1^ Department of Orthopedic Surgery and Sports Medicine Center, The First Affiliated Hospital and The First Clinical College, Jinan University, Guangzhou, China; ^2^ Pediatric Cardiac Surgery Center, National Center for Cardiovascular Disease and Fuwai Hospital, Chinese Academy of Medical Sciences, Peking Union Medical, Beijing, China; ^3^ Department of Breast Surgery, Peking Union Medical College Hospital, Chinese Academy of Medical Science and Peking Union Medical College, Beijing, China; ^4^ Department of General Surgery, The First Affiliated Hospital of Jinan University, Guangzhou, China

**Keywords:** pharmacological mechanisms, tendon injury, tendon rupture, tendon healing, tendon adhesion, Shujin Huoxue tablet

## Abstract

Tendon impairment is a common injury associated with impairment of range of motion and pain. Currently, evidence has confirmed that natural herbs contribute to orthopedics and have shown excellent results in the clinical management of tendon impairment. Shujin Huoxue tablet (SHT) and its complex prescriptions are regularly used in tendon rupture therapy with positive results. This study aimed to discover the potential molecules that promote tendon healing. The Chinese traditional medicine system pharmacological database analysis platform (TCMSP) is the primary resource. The Traditional Chinese Medicine Integrated Database and Encyclopedia of Traditional Chinese Medicine database were used as secondary databases. The GeneCards database was used to search for reported tendinopathy-related genes by keywords. Functions of the targeted genes were analyzed using Gene Ontology enrichment analysis and Kyoto Encyclopedia of Genes and Genomes. Protein–protein interaction information was extracted from the STRING database. Docking study, MTT assay, quantitative real-time PCR, and migration assays were performed to obtain a better understanding of the herbs according to cell function to test the basic pharmacological action *in vitro*. A total of 104 disease nodes, 496 target gene nodes, 35 ingredient nodes, and one drug node were extracted. According to the TCMSP database, 6-hydroxykaempferol, which reportedly promotes the proliferation of microvascular endothelial cells, is a molecule found in SHT. We found that it promoted the proliferation and migration of tendon fibroblasts and elevated tendon repair-related gene expression. Purified 6-hydroxykaempferol promoted the proliferation and migration of tendon fibroblasts and increased their mRNA expression in tendon proliferation.

## Introduction

Tendon injuries, such as pain and lack of mobility, are common in both athletes and nonathletes (Watts et al., 2017). In modern medicine, oral nonsteroidal anti-inflammatory drugs (NSAIDs) are regularly used to manage tendon injuries (Jones et al., 2015). However, NSAIDs injure the mucosa of the stomach and duodenum. The primary mechanism of gastrointestinal mucosal injury theory is NSAID-induced inhibition of mucosal prostaglandin synthesis (Shim and Kim, 2016).

Currently, evidence confirms that natural herbs contribute to orthopedics with excellent results in clinical management (Lo et al., 2019). Shujin Huoxue tablet (SHT) and its complex prescriptions are frequently used in tendon rupture therapy, with promising results (Guosen and Washing, 2014; Tseng et al., 2018). Network pharmacology combines various biological and drug databases as an up-to-date model for drug research. It constructs a network prediction model of drug–gene–target protein–disease, resulting from bioinformatics analyses, to predict drug action targets and analyze drug action mechanisms from the perspective of biological network balance (Zhao et al., 2015; Fang et al., 2017; Lyu et al., 2017). However, research on purified molecules of SHT compound prescriptions is limited. This study aimed to discover the potential molecules that promote tendon healing. Furthermore, this study aimed to deliver a theoretical basis for further experimental studies and rational clinical applications of SHT based on pharmacological validation.

## Materials and Methods

### Major Chemical Components of Shujin Huoxue Tablet

**FIGURE 1:**
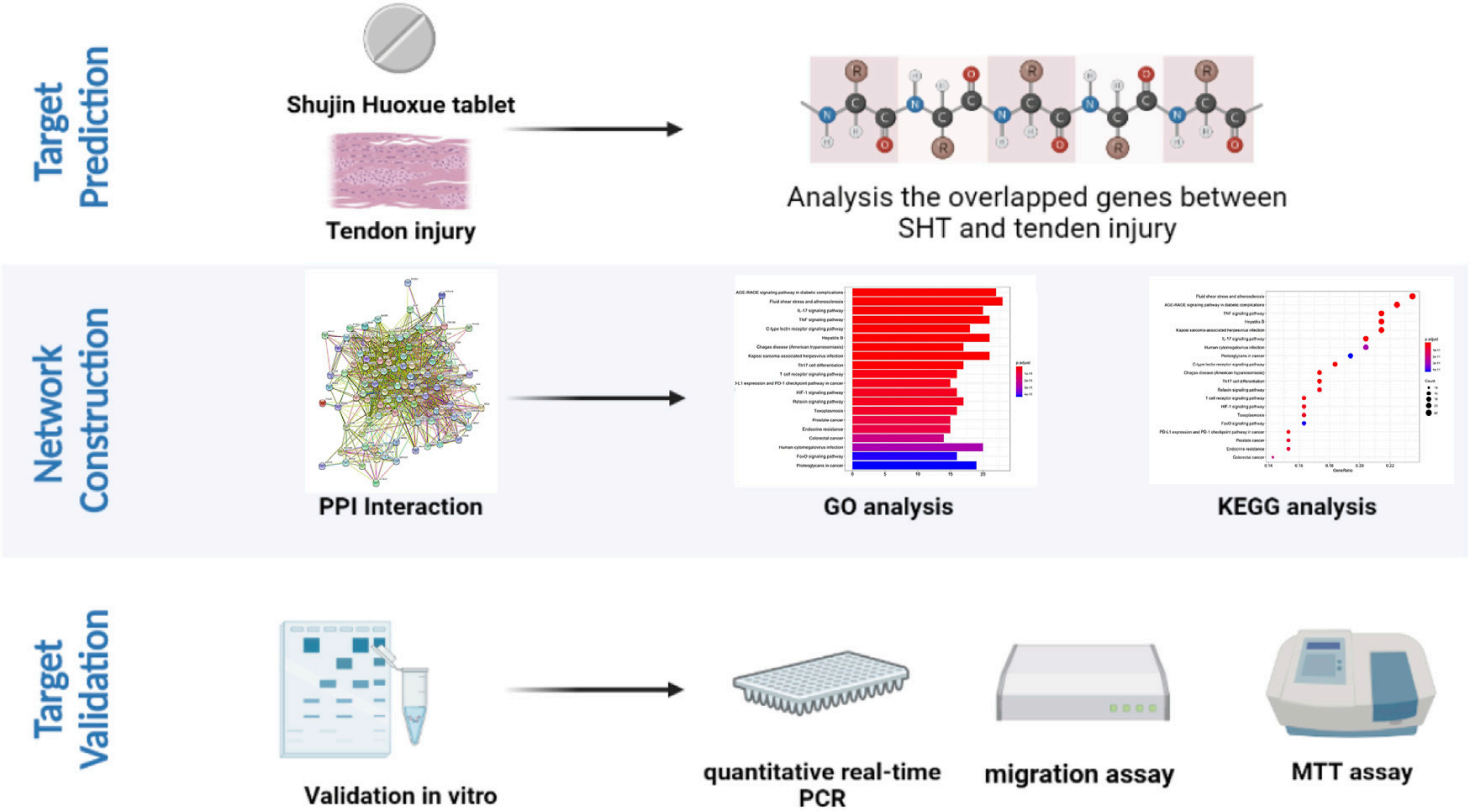
Flow chart of the research strategy created with BioRender.com.

The Chinese traditional medicine system pharmacological database analysis platform (TCMSP) was used as the primary database in this study. Moreover, the Traditional Chinese Medicine Integrated Database (TCMID) and Encyclopedia of Traditional Chinese Medicine (ETCM) database were used as secondary databases. These databases were used in combination to bridge any gaps in key information, such as the name or number of ingredients. Even with the same database and screening strategy, the components included in the analysis vary (Ming, 2021). Therefore, all the data from the databases were integrated. Literature mining and the GoPubMed platform were used to confirm the molecular structure, using the mol2 file format for the components (Figure 1). The database uses the HIT database prediction algorithm, SysDT, to identify the relationship between drug targets. Disease information in this database was obtained from the TTD and PharmGKB databases. The value of TCMSP is that it provides pharmacokinetic information for each compound, such as drug product similarity (DL), oral bioavailability (OB), potential to cross the blood–brain barrier (BBB), intestinal epithelial permeability (Caco-2), lipid–water partition coefficient (ALogP), and the number of H-bond donor/acceptor (Hdon/Hacc). Therefore, users can select compounds with desirable absorption, distribution, metabolism, and excretion (ADME) characteristics for further studies (Ru et al., 2014).

TCMID collects TCM-related information from different sources through text mining. Furthermore, it links common drug and disease databases, including DrugBank, OMIM, and PubChem. Chemical changes may occur during the torment of prescriptions, resulting in the generation of new ingredients. Data containing prescription ingredients were collected from the website, and 778 herbal mass spectra (MS) related to 170 herbs were added. Spectral data were analyzed to show the variation in the quality of herbal medicines from different sources and to differentiate between genuine medicinal materials and common medicinal materials. A massive increase in website analysis data will facilitate the study of combination therapy and the understanding of the underlying mechanisms of TCM at the molecular level (Huang et al., 2018).

ETCM data on 402 herbs, 3,959 TCM compounds, 7,284 TCM chemical constituents, 2,266 drug targets, and 4,323 related diseases were collected. Data collected on herbal medicines included details of their origin, medicinal taste (sour, bitter, sweet, pungent, and salty), medicinal properties (cold, hot, warm, cool, and flat), return meridians (lung meridian and liver meridian), indications, ingredients, and quality control standards. Furthermore, other information such as compound name, dosage form, composition, indications, and ingredients, including the molecular formula, molecular weight, various physical and chemical indicators, ADME parameters, and drug-like properties at the compound level, was collected. Quantitative standards were set according to the Pharmacopoeia of the People’s Republic of China (2015 version) which are the official TCM quality evaluation standards in China (Xu et al., 2019).

The absorption, distribution, metabolism, and excretion (ADME) processes of the active compounds are examined. In this process, the oral bioavailability (OB), drug similarity (DL), and half-life (HL) were the crucial parameters of ADME. The active compounds are indicated with OB ≥ 30% and DL ≥ 0.18 (Figure 1).

### Prediction of Potential Targets

The GeneCards (RRID:SCR_002773) database was used to search for reported tendinopathy-related genes by entering keywords of tendon injuries, tendon rupture, tendon healing, and tendon adhesion for target-based drug applications. The potential tendon-healing targets of the active components of SHT matched the potential gene targets of the active ingredients of SHT. According to the UniProt database (https://www.uniprot.org/), the full names of the target proteins were transformed into gene symbols based on the UniProt ID (The UniProt Consortium, 2017) (Figure 1). The database leverages the unique rich combinatorial annotations of GeneCards. The GeneCards database provides direct links to gene-related research reagents, such as antibodies, recombinant proteins, DNA clones, and inhibitory RNAs, and lists of gene-related drugs and compounds. GeneCards was used to depict the GeneCards Inferred Functionality Score annotation landscape tool for scoring the functional status of genes (Fishilevich et al., 2016).

### Analysis of Functional Enrichment

Various biological processes and molecular functions were provided through the Database for Annotation, Visualization, and Integrated Discovery (DAVID, https://david.ncifcrf.gov/) v6.8. The examination of the function target genes based on DAVID was carried out through the Gene Ontology (RRID:SCR_002811) biological process (GOBP) enrichment analysis and Kyoto Encyclopedia of Genes and Genomes (KEGG) pathway enrichment analysis. A *p*-value of 0.05 or less represents importance in the analysis. The capabilities provided by DAVID accelerate the analysis of genome-scale datasets by facilitating the transition from data collection to biological meaning, and the analysis results and graphical displays remain dynamically linked to raw data and external data repositories, providing in-depth and extensive data cover.

### Network Pharmacology Evaluations

In network pharmacology, it is necessary to evaluate the reliability, normativeness, and rationality of the data collected. The specific requirements include three steps. First, in the reliability evaluation, the reliability of acquisition of main data and its associated information, the design of software algorithms and analysis methods, and the selection of verification methods and model building were evaluated according to their ability to meet the analysis requirements. Second, in the normative evaluation, the process of data information extraction and conversion, software/algorithm development, network construction, and analysis were evaluated. Moreover, the experimental verification is standardized, and the accuracy of relevant technical methods is evaluated to ensure the accuracy of the analysis results and reproducibility. Finally, during rationality evaluation data screening and filtering, selection of network analysis indicators and determination of thresholds, and selection of verification models and detection indicators were evaluated for reasonability (Li, 2021).

### Docking Study

The molecular structure of active ingredients in the mol2 format was obtained from the TCMSP database. The PDB format of the 3D molecular structure of the corresponding target (protein) gene was obtained from the PDB (https://www.rcsb.org) database. The molecular structure documents of active ingredients and key target genes were imported into AutoDock (RRID:SCR_012746) Tools 1.5.6 software for molecular docking, and the documents in the PDBQT format were imported into Open Babel software and converted them into the PDB format. PyMOL 2.5.0 software was used for visual analysis of the target protein and the compound with a high docking score and relatively stable conformation.

### Cell Culture

Primary rat tendon fibroblasts were purchased from ProCell in Wuhan, China (CP-R237). According to the manufacturer’s instructions, cells were digested with 0.25% trypsin and seeded in six-well plates, with 2 ml culture medium made of Dulbecco’s modified Eagle’s medium with 10% fetal bovine serum (FBS), 100 U/ml penicillin, and 100 mg/ml streptomycin in each well and incubated at 37°C in a humidified atmosphere of 5% CO_2_: 95% air. When cell confluence reached 90%, the cells were subcultured by trypsinization at a 1:6 dilution ratio. Cells from passage 3 were used for subsequent experiments.

### MTT Assay

Tendon fibroblasts (3 × 10^3^) were seeded in each well of a 96-well culture plate with culture medium containing DMEM (0.1 ml of DMEM), 10% FBS, 100 U/ml penicillin, and 100 mg/ml streptomycin in each well. Then, 6-hydroxykaempferol (Shanghai Yuanye Bio-Technology Co., Ltd., B50362) was added to each well at concentrations of 50, 100, 200, and 500 μg/ml. After incubation at 37°C for 24 h, cells were washed once with 1× phosphate-buffered saline (PBS), followed by adding 0.1 ml DMEM with 0.1 ml 3-(4,5-dimethyl-2-thiazolyl)-2,5-diphenyl-2-H-tetrazolium bromide (MTT, Servicebio, G4101-200T). After incubation at 37°C for 30 min, the media were removed, and formazan crystals in the cells were solubilized in 0.2 ml DMSO and processed for OD reading at 570 nm using a spectrophotometer. Five wells were used for each concentration for each experiment, and the entire experiment was repeated three times.

### RNA Extraction, RT-PCR, and Quantitative Real-Time PCR

Total cellular RNA was extracted using a miRNeasy Mini Kit (Qiagen, 217004) according to the manufacturer’s instructions. Total RNA (2 μg of total RNA was reverse-transcribed to cDNA using the PrimeScript reverse transcription system (TaKaRa, 2680A). Quantification of the RNA levels of *Col1a1* and *Tenascin C* (*TNC*) was achieved by quantitative real-time PCR using an ABI ViiA7 system and PowerUp SYBR Green Master Mix (Applied Biosystems, A25742). The data were normalized to the GAPDH expression. The PCR program consisted of 95°C for 5 min, 40 cycles of 95°C for 30 s, 56–58°C for 30 s, and 72°C for 30 s, followed by a final extension step at 72°C for 10 min. All reactions were conducted in triplicate. The data were analyzed using the threshold cycle (Ct) method. The primer sequences for the target genes were as follows: *Col1a1* forward primer, 5′-CCC​AGC​GGT​GGT​TAT​GAC​TT-3'; *Col1a1* reverse primer, 5′- TCG​ATC​CAG​TAC​TCT​CCG​CT-3'; *TNC* forward primer, 5′- CAG​AGT​TGC​CAC​CTA​CTT​GCC-3'; *TNC* reverse primer, 5′-TCT​CTC​CCT​CAT​CTT​CTT​TGT​TCA-3'; *GAPDH* forward primer, 5′-CTG​GAG​AAA​CCT​GCC​AAG​TAT​G -3′; and *GAPDH* reverse primer, 5′-GGT​GGA​AGA​ATG​GGA​GTT​GCT-3'.

### Migration Assay

A scratch wound assay was performed to test the migration of tendon fibroblasts. Tendon fibroblasts (1 × 10^6^) were seeded in each well of a six-well culture plate with culture medium containing 2 ml of DMEM with 10% FBS, 100 U/ml penicillin, and 100 mg/ml streptomycin in each well and cultured overnight at 37°C. After cell confluency reached over 95%, the medium was removed, and the cells were washed once with 1× PBS. Then, a linear scratch within the cell monolayer was generated using a sterile 1,000-μl plastic pipette tip. The cellular debris was washed with 1× PBS. Next, 2 ml starvation medium composed of DMEM with 1% FBS, 100 U/ml penicillin, and 100 mg/ml streptomycin was added to each well, followed by treatment with 400 μg of 6-hydroxykaempferol in three wells, and the cells were incubated at 37°C to allow migration. At 0 and 3 h, the images were photographed (10× magnification). The percentage of the open wound area (3 h relative to 0 h) was calculated using ImageJ software.

### Statistical Analysis

Data are the mean ± SD. The significance of the results was determined based on one-way analysis of variance using Prism 8.0.1 (GraphPad Prism (RRID:SCR_002798), San Diego, CA, United States). *p* < 0.05 was considered significant and presented with * in the figure.

## Results

### Network Pharmacology Evaluation

The evaluation group was established with a clear analysis of objects and goals. The evaluation content was then determined. The evaluation implementation achieved a general and scalable evaluation. The results indicated that the evaluation was qualified.

### Target Genes of Shujin Huoxue Tablet Prescription

According to the TCMSP, TCMID, and ETCM databases, the SHT contains a total of 104 species. Species that could be identified include Carthami flos with 22 species, Cyperi Rhizoma with 18 species, Cortex Periplocae Radicis with 17 species, *Trachelospermum jasminoides* with 9 species, Rhizome of whiteback greenbrier with 5 species, Lycopi Herba with 2 species, *Viscum angulatum* heyne with 7 species, and *Spatholobus suberectus* Dunn with 24 species.

### Disease-Related Gene Extraction

Considering that the SHT prescription is used for killing pain, setting bone, and alleviating arthritis, the disease is related to the tendon injury, adhesion, and healing. The genes overlapped the disease-related genes, and the target genes of active compounds were pooled. Then, 104 genes were covered from tendon injury.

### Validation of Purified Molecules

**FIGURE 2:**
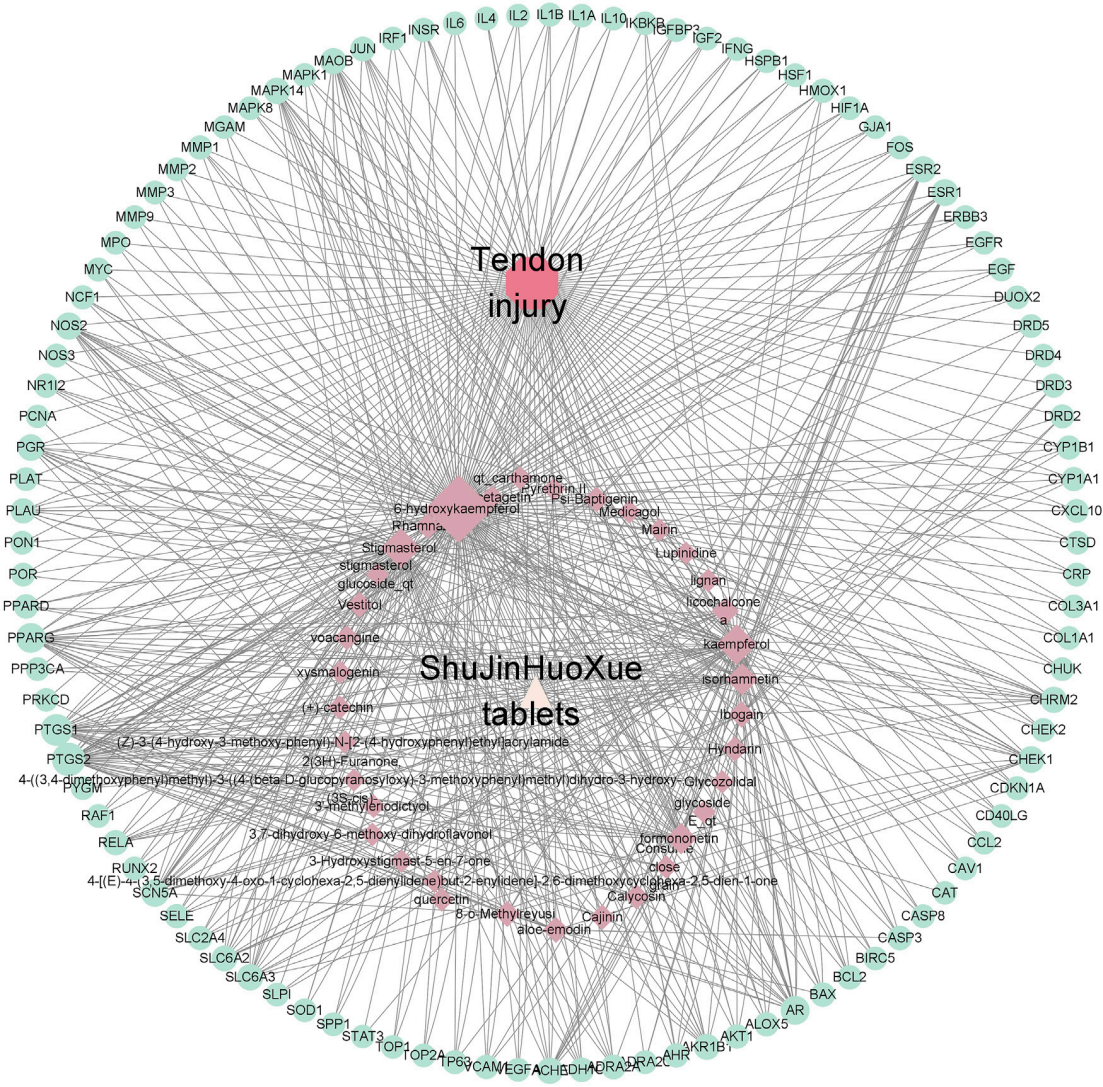
Ingredient–target genes–pathway network of tendon injury.

In terms of tendon injury, 673 genes were in total in the network. Then, 104 disease nodes, 496 target gene nodes, 35 ingredient nodes, and 1 drug node were extracted. According to the result, the data processed by STRING (version 11.0) were used to produce a PPI network. It shows the protein–protein interaction between the disease, including tendon injury, tendon adhesion and tendon healing, and the target gene of the active compound. The simple tabular text is extracted from the graphic to run the R program (version 3.6.2). From Figure 4, AKT 1 showed a high degree of coreness with 79 times interaction. IL6, CASP3, and VEFGA followed AKT 1 with 71, 68, and 68 times of interaction, respectively. The more interaction indicated the higher chance to become core proteins (Figure 2).

### Functional Annotation Analysis

The overlapped genes were further studied through GO-BP and KEGG (RRID:SCR_012773) enrichment analysis. As shown in Figure 1, those genes were significantly related with KEGG pathway of “AGE-RAGE signaling pathway in diabetic complications” (*p* = 2.88E-22), “fluid shear stress and atherosclerosis” (*p* = 2.94E-20), “IL-17 signaling pathway” (*p* = 5.69E-20), and “TNF signaling pathway” (*p* = 1.02E-19) in the healing process (Supplementary Table S2). In terms of GO-BP analysis, “nuclear receptor activity” (*p* = 2.63E-12), “transcription factor activity, direct ligand-regulated sequence-specific DNA binding” (*p* = 2.63E-12), “cytokine receptor binding” (*p* = 3.31E-10), “tetrapyrrole binding” (*p* = 5.01E-09), “cytokine activity” (*p* = 1.97E-09), “heme binding” (*p* = 2.50E-09), “steroid hormone receptor activity” (*p* = 1.78E-08), “receptor ligand activity” (*p* = 2.43E-07), “DNA-binding transcription activator activity, RNA polymerase II-specific” (*p* = 5.48E-07), “growth factor receptor binding” (*p* = 5.64E-07), and “scaffold protein binding” (*p* = 6.86E-07) were significantly enriched in the healing process (Supplementary Table S3).

### Validation of Purified Molecules

**FIGURE 3:**
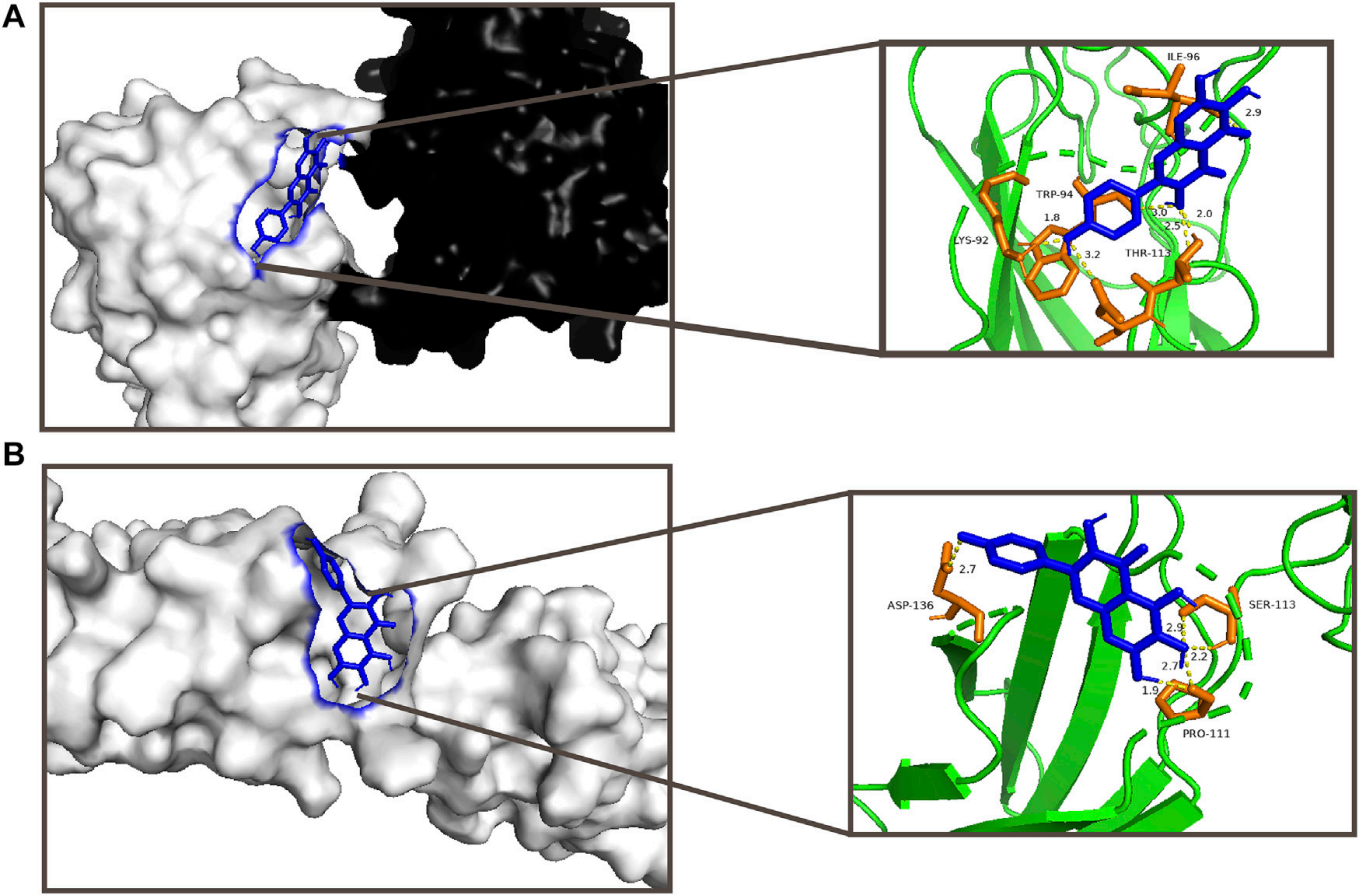
Visual analysis of compounds with higher scores and more stable conformation and target proteins by the docking study; **(A)** interaction between 6-hydroxykaempferol and *Col1a1* with an affinity of −4.63 kcal/mol; **(B)** interaction between 6-hydroxykaempferol and *TNC* with an affinity of −5.21 kcal/mol.

**FIGURE 4:**
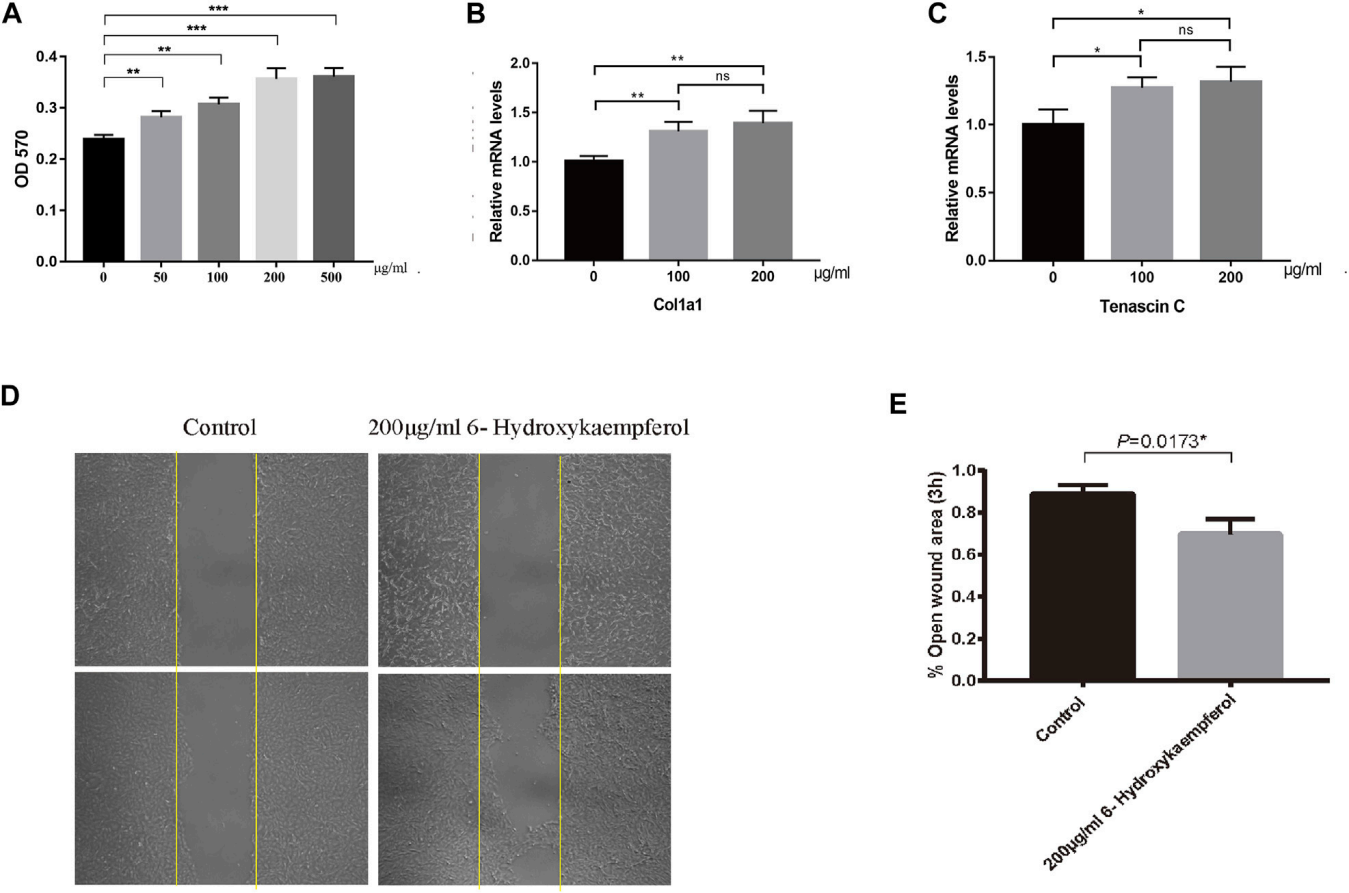
6-Hydroxykaempferol was evaluated as a fibroblast proliferator. **(A)** Tendon fibroblasts (3 × 10^3^) were seeded in each well of a 96-well culture plate for MTT assay. Quantification of the RNA levels for **(B)** *Col1a1* and **(C)** *TNC* was achieved using quantitative real-time PCR. **(D)** Scratch wound assay was performed to test the migration of tendon fibroblasts. **(E)** Percentage of the open wound area.

According to the TCMSP database, 6-hydroxykaempferol is a molecule found in SHT (Ru et al., 2014). Several studies have shown that 6-hydroxykaempferol promotes the proliferation of microvascular endothelial cells (Yao et al., 2016; Liao et al., 2018). The positive results include the highest DC values among 6-hydroxykaempferol for docking study affinity values of −4.63 kcal/mol in *Col1α1* and −5.21 kcal/mol in *TNC* (Figure 3). Then, 6-hydroxykaempferol was therefore chosen for validation. This is the first study to explore the effects of this particular molecule on tendon fibroblasts. Since tendon repair is highly related to fibroblast proliferation, migration, and differentiation, the following molecular analyses were carried out to establish the potential relationship between 6-hydroxykaempferol and tendon repair (Chang et al., 2011; Guo et al., 2019). Analysis of its effects on cell viability showed that the herb improved proliferation in a concentration-dependent manner. Hierarchically organized collagen fibrils in the extracellular matrix (ECM) form tendons. The gene *Col1α1* encodes type I collagen, which has the highest percentage of collagen fibrils (Guerquin et al., 2013). In contrast, *TNC* participates in the regulation of tendon repair (Xu et al., 2021), and its expression significantly increases during tendon-derived stem cell-mediated tendon repair. Therefore, these two genes were chosen for expression analyses, and positive results were obtained for both *Col1a1* and *TNC*. Furthermore, a migration assay showed that 6-hydroxykaempferol at a concentration of 200 μg/ml promoted tendon fibroblast migration. The percentage of open wound area significantly decreased at 3 h after treatment (*p* = 0.0173). Our results showed that treatment with 6-hydroxykaempferol was able to promote tendon fibroblast proliferation, highly related gene expression, and migration (Figure 4).

## Discussion

The treatment strategy for tendon injury is a clinical challenge. Furthermore, the basic mechanisms of intratendinous and tendon-to-bone healing remain only partially understood (Thomopoulos et al., 2015). Full coordination between the intrinsic tendon core tissue and surrounding extrinsic synovial tissues is needed for tendon tissue repair. According to a recent study, metabolic demands on resident tendon cells may be crucial for the regulation of tissue compartments involved in tendon recovery. SHT, a Chinese medicine consisting of herbal prescriptions widely used in clinical practice in China, can relax muscles and stimulate blood circulation (Tong et al., 2012), which may help metabolic demands in tendon repair. Our study provides evidence consistent with this theory. Moreover, 6-hydroxykaempferol is a competitive inhibitor of tyrosinase (Information NCIB, 2022). Naturally occurring flavonoids from a set of 14 hydroxy-flavones tested were previously shown to exhibit high inhibitory effects on tyrosinase compared to L-DOPA (Gao et al., 2007). However, this has never been studied in tendon reconstructions.

Previous studies have reported the anti-platelet aggregation, anticoagulation, and antioxidation effects of 6-hydroxykaempferol (Yao et al., 2016). Moreover, it has been reported to promote blood circulation to remove blood stasis, which may explain its anti-inflammatory effects, pain relief, muscle relaxation, and increased blood flow. Therefore, it advances the tendon repair process, which is consistent with the experimental results. This effect is believed to be achieved by cumulative biological exposure to antioxidants, which can quench the proliferation of radical oxygen and reactive nitrogen species, which are implicated in the pathology of fibroblasts (Wootton-Beard et al., 2011).

## Strengths and Limitations

This is the first study to explore the characteristics of 6-hydroxykaempferol on tendon repair. To prove this variability, MTT assays, quantitative real-time PCR, and migration assays were performed to test the basic pharmacological action. Network pharmacology mainly relies on the integrated analysis of data from different sources, and the emergence of massive data has brought new challenges to the development of network pharmacology. Specifically, it has three main aspects. First, the data sources are scattered, and integration is difficult. Databases are the main data sources of network pharmacology, but there are challenges, such as large differences in data collection in different databases and relatively independent data. Second, the data usage has not been standardized. Owing to the lack of systematic research on the impact of data from different sources on the analysis results and the applicability of different databases, irregularities in the use of various types of data are inevitable. Third, *in vitro* cell verification was performed, but the cell experiments did not fully reflect the overall effect of the compound. However, different verification methods have certain limitations. According to the guidelines, the overall efficacy of the drug was clinically verified. Subsequently, the specific mechanism of action or target was further verified (Li. 2021).

Clinically, SHT has been used to treat tendon injury. However, a molecular validation of its effects has not previously been conducted. Based on *ex vivo* experiments, 6-hydroxykaempferol is able to promote tendon fibroblast proliferation, high mRNA expression (in *Col1A1* and *TNC*), and migration in tendon proliferation. Further studies should be conducted to determine the potential characteristics of 6-hydroxykaempferol.

## Data Availability

The original contributions presented in the study are included in the article/Supplementary Materials; further inquiries can be directed to the corresponding author.
