# Supplementary material for: Effects of 6-Hydroxykaempferol: A Potential Natural Product for Amelioration of Tendon Impairment
Source: Front Pharmacol. 2022 Jul 22;13:919104. doi: 10.3389/fphar.2022.919104 (PMC9354238; doi:10.3389/fphar.2022.919104)
Supplement: Supplementary file 1 [file Table1.DOCX]

| Supplemental Table 1 Molecular List | | |
| --- | --- | --- |
| Molecular Name | ID | Structure |
| 4-[(E)-4-(3,5-dimethoxy-4-oxo-1-cyclohexa-2,5-dienylidene)but-2-enylidene]-2,6-dimethoxycyclohexa-2,5-dien-1-one | MOL002694 | 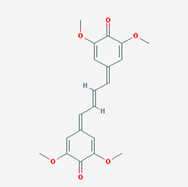 |
| lignan | MOL002695 | 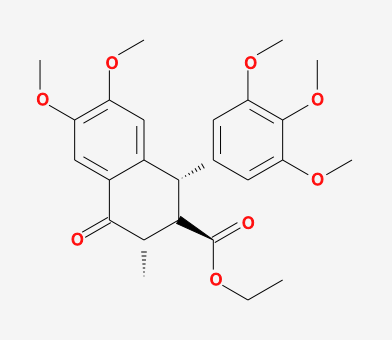 |
| pyrethrin II | MOL002710 | 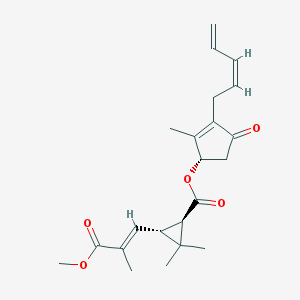 |
| 6-Hydroxykaempferol | MOL002712 | 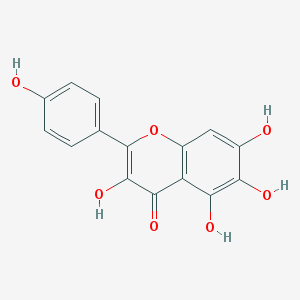 |
| qt_carthamone | MOL002717 | 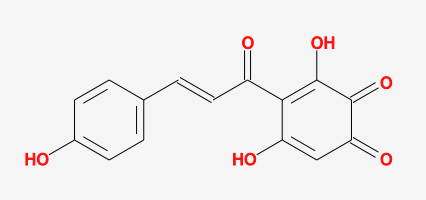 |
| quercetagetin | MOL002721 | 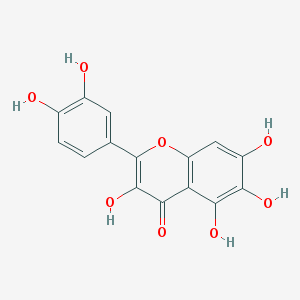 |
| kaempferol | MOL000422 | 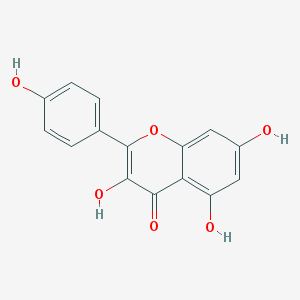 |
| stigmasterol | MOL000449 | 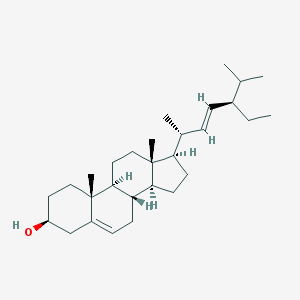 |
| quercetin | MOL000098 | 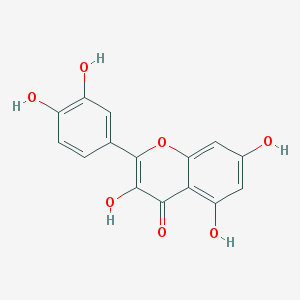 |
| isorhamnetin | MOL000354 | 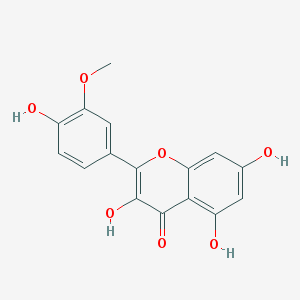 |
| Hyndarin | MOL004071 | 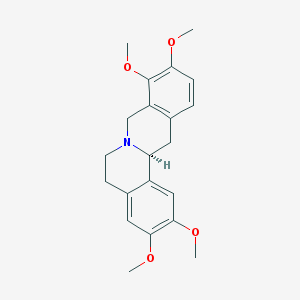 |
| stigmasterol glucoside_qt | MOL004074 | 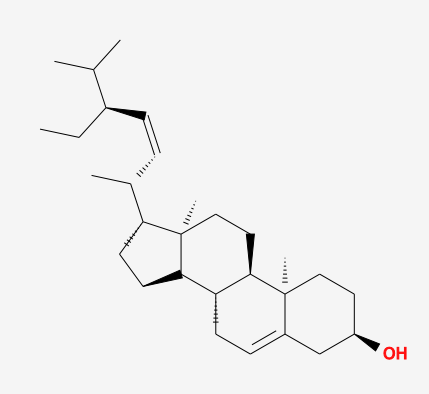 |
| Glycozolidal | MOL005656 | 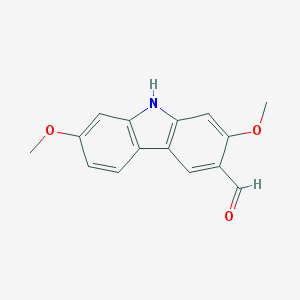 |
| glycoside E_qt | MOL005664 | 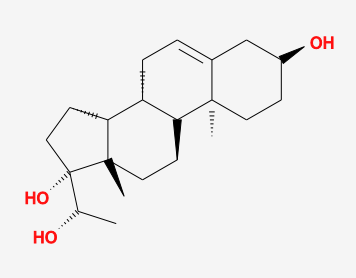 |
| xysmalogenin | MOL005693 | 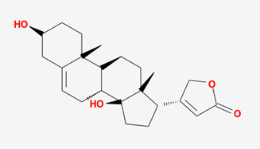 |
| Ibogain | MOL000529 | 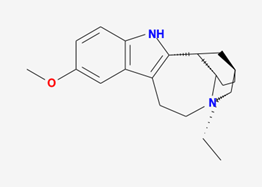 |
| Mairin | MOL000211 | 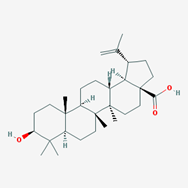 |
| 3'-methyleriodictyol | MOL000338 | 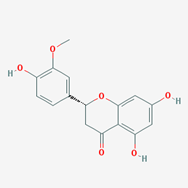 |
| Rhamnazin | MOL000351 | 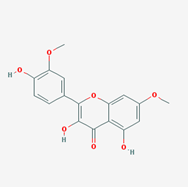 |
| Calycosin | MOL000417 | 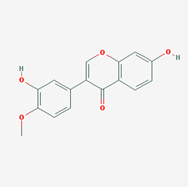 |
| Canin | MOL000502 | 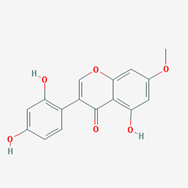 |
| Medicagol | MOL000503 | 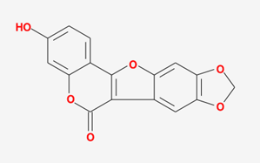 |
| Lupinidine | MOL000506 | 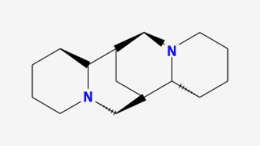 |
| Psi-Baptigenin | MOL000507 | 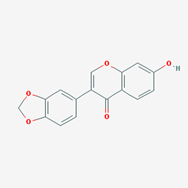 |
