# Supplementary material for: Effects of 6-Hydroxykaempferol: A Potential Natural Product for Amelioration of Tendon Impairment
Source: Front Pharmacol. 2022 Jul 22;13:919104. doi: 10.3389/fphar.2022.919104 (PMC9354238; doi:10.3389/fphar.2022.919104)
Supplement: Supplementary file 2 [file Table2.pdf]

Supplemental Table 2 KEGG analysis

ID	Description	GeneRatio	pvalue	qvalue	Count
**hsa04933**	AGE-RAGE signaling pathway in diabetic complications	11/49	2.88E-22	2.25E-20	22
**hsa05418**	Fluid shear stress and atherosclerosis	23/98	2.94E-20	1.14E-18	23
**hsa04657**	IL-17 signaling pathway	10/49	5.69E-20	1.48E-18	20
**hsa04668**	TNF signaling pathway	3/14	1.02E-19	1.98E-18	21
