# Supplementary material for: Effects of 6-Hydroxykaempferol: A Potential Natural Product for Amelioration of Tendon Impairment
Source: Front Pharmacol. 2022 Jul 22;13:919104. doi: 10.3389/fphar.2022.919104 (PMC9354238; doi:10.3389/fphar.2022.919104)
Supplement: Supplementary file 3 [file Table3.pdf]

Supplemental Table 3 GO analysis

ID	Description	GeneRatio	pvalue	qvalue	Count
GO:0004879	nuclear receptor activity	9/94	2.63E-12	2.96E-10	9
GO:0098531	transcription factor activity, direct ligand regulated sequence-specific DNA binding	9/94	2.63E-12	2.96E-10	9
GO:0005126	cytokine receptor binding	7/47	3.31E-10	2.48E-08	14
GO:0005125	cytokine activity	12/94	1.97E-09	1.11E-07	12
GO:0020037	heme binding	10/94	2.50E-09	1.13E-07	10
GO:0046906	tetrapyrrole binding	10/94	5.01E-09	1.88E-07	10
GO:0003707	steroid hormone receptor activity	7/94	1.78E-08	5.74E-07	7
GO:0048018	receptor ligand activity	7/47	2.43E-07	6.85E-06	14
GO:0001228	DNA-binding transcription activator activity, RNA polymerase II-specific	13/94	5.48E-07	1.27E-05	13
GO:0070851	growth factor receptor binding	8/94	5.64E-07	1.27E-05	8
GO:0097110	scaffold protein binding	6/94	6.86E-07	1.41E-05	6
